# The Use of Induced Pluripotent Stem Cells as a Model for Developmental Eye Disorders

**DOI:** 10.3389/fncel.2020.00265

**Published:** 2020-08-20

**Authors:** Jonathan Eintracht, Maria Toms, Mariya Moosajee

**Affiliations:** ^1^UCL Institute of Ophthalmology, London, United Kingdom; ^2^The Francis Crick Institute, London, United Kingdom; ^3^Moorfields Eye Hospital NHS Foundation Trust, London, United Kingdom; ^4^Great Ormond Street Hospital for Children NHS Foundation Trust, London, United Kingdom

**Keywords:** eye development, human induced pluripotent stem cells, developmental eye disorders, disease modeling, ocular maldevelopment, *VSX2*, microphthalmia, corneal hereditary endothelial dystrophy

## Abstract

Approximately one-third of childhood blindness is attributed to developmental eye disorders, of which 80% have a genetic cause. Eye morphogenesis is tightly regulated by a highly conserved network of transcription factors when disrupted by genetic mutations can result in severe ocular malformation. Human-induced pluripotent stem cells (hiPSCs) are an attractive tool to study early eye development as they are more physiologically relevant than animal models, can be patient-specific and their use does not elicit the ethical concerns associated with human embryonic stem cells. The generation of self-organizing hiPSC-derived optic cups is a major advancement to understanding mechanisms of ocular development and disease. Their development *in vitro* has been found to mirror that of the human eye and these early organoids have been used to effectively model microphthalmia caused by a *VSX2* variant. hiPSC-derived optic cups, retina, and cornea organoids are powerful tools for future modeling of disease phenotypes and will enable a greater understanding of the pathophysiology of many other developmental eye disorders. These models will also provide an effective platform for identifying molecular therapeutic targets and for future clinical applications.

## Introduction

Developmental eye disorders are amongst the most common cause of severe visual impairment in children, with a combined incidence of 1–2 per 10,000 births (Nedelec et al., 2019). They comprise a wide range of congenital abnormalities ranging from anophthalmia, aniridia, Leber congenital amaurosis, and congenital cataracts, and are frequently associated with extraocular features (Bardakjian et al., 2015). Childhood blindness can have extensive ramifications for the child and their family, particularly as the global cost is higher than adult-onset vision loss (Rahi et al., 1999; Dharmasena et al., 2017). Quality of life, educational opportunities, mental health, and independence are all affected by sight loss (Tseng and Coleman, 2018). Currently, there are no preventative strategies, and management is only supportive to maximize any residual vision and minimize amblyopia. Ocular malformations can result from several environmental factors, including exposure to teratogenic drugs or maternal infections; however, it has been estimated that genetic variants are responsible for approximately 80% of cases (Gregory-Evans et al., 2019). Approximately 70% of patients with a bilateral or severe phenotype will receive a molecular diagnosis but 90% with a unilateral phenotype remain unresolved (Harding and Moosajee, 2019). The early *in utero* onset of these diseases poses a challenge for investigating underlying genetic mechanisms and developing suitable treatments.

There is a diverse range of developmental eye disorders, which can vary depending on the stage of development, genetic pathways and tissue(s) affected (Gregory-Evans et al., 2019). Additionally, there is large phenotypic and genetic heterogeneity within the same group of diseases (Williamson and FitzPatrick, 2014). One of the major early-onset disease groups arising between weeks 4–7 of gestation is the microphthalmia/anophthalmia/coloboma (MAC) spectrum, which varies in severity and includes the complete absence of an eye (anophthalmia), a small underdeveloped eye (microphthalmia) and incomplete fusion of the optic fissure leading to a persistent cleft in the inferior aspect of the eye spanning one or more of the following tissues: iris, ciliary body, retina, RPE, choroid and optic nerve (coloboma; Harding and Moosajee, 2019). All these disorders are caused by disruption to key regulatory genes, including numerous transcription factors, that are essential for normal eye development (Moosajee et al., 2018). By understanding the roles of these genes in development, the pathological mechanisms and phenotypic variation can be better understood, improving diagnosis and management.

## A New Tool to Study Ocular Development

Animal models, including the mouse, rat, zebrafish, *drosophila, Xenopus*, chick and dog have all contributed to our understanding of ocular development and disease (Kaukonen et al., 2018; Kolosova et al., 2018; Moore et al., 2018; Sghari and Gunhaga, 2018; Zhu et al., 2018; Kha et al., 2019; Richardson et al., 2019). Despite their invaluable contribution, animal models are suboptimal for critical reasons: (i) Differences in gene expression between animal models do not inform our understanding of human disease mechanisms; for example, *MAB21L2*, which is required for eye morphogenesis and cell survival in the developing optic cup and lens, and is associated with microphthalmia and coloboma in humans (Gath and Gross, 2019; Eintracht et al., 2020). However, the closest expression pattern to humans is still unknown due to differing *mab21l2* expression patterns and localization in the chick, mouse, and zebrafish (Sghari and Gunhaga, 2018; Gath and Gross, 2019). (ii) Disease phenotypes observed in humans do not always mimic those seen in animals; for instance, heterozygous *PITX3* mutations in humans primarily result in dominant anterior segment dysgenesis and cataracts but homozygous loss-of-function mutations result in microphthalmia in mice (Rosemann et al., 2010; Ma et al., 2018a). (iii) The embryonic lethality described in many animal models e.g., *Sox2, Otx2*, and *Mab21l2* mouse and zebrafish models is incomparable (Reis and Semina, 2015). (iv) Ocular structures and developmental events differ between humans and animal models as highlighted in zebrafish, where the optic vesicles are solid neuroepithelial protrusions from the cell-dense neural tube (neural keel) that then cavitate, whereas human optic vesicles are hollow (Richardson et al., 2017). (v) The macula is not present in rodent eyes, thus disease pathophysiology differs greatly to human disorders affecting the central retina (Huber et al., 2010).

As a result, studying ocular development and disease using human tissue is more physiologically relevant. However, understanding mechanisms of early ocular malformations using human samples is near-impossible due to the inaccessibility to fetal tissue from 4 to 7 weeks of gestation (Lindsay et al., 2016). Consequently, the use of human-induced pluripotent stem cells is an attractive option to overcome these difficulties.

## Human Induced Pluripotent Stem Cells

Human-induced pluripotent stem cells (hiPSCs) are generated from somatic cells by delivery of the “Yamanaka” factors, *OCT4, SOX2, KLF-4*, and *C/L-MYC* (Takahashi et al., 2007; Okita et al., 2011). Overexpression of these transcription factors will activate endogenous gene expression regulating pluripotent gene expression (Black and Gersbach, 2018). Consequently, cells will revert to a pluripotent state in terms of morphology, proliferation, gene expression, epigenetics, and differentiation capacity (Takahashi et al., 2007). Morphological and molecular similarities between hiPSCs and human embryonic stem cells (hESCs) have been extensively demonstrated and recent data suggest they cannot be distinguished by a unique and consistent gene expression signature (Choi et al., 2015). Further comparisons of hESC- and hiPSC-derived neurons revealed that epigenetic and gene expression profiles are remarkably similar (de Boni et al., 2018).

While it is still unknown as to what extent hiPSCs can entirely replace hESCs due to the unique genetic signature contained in each line, it is important to note the distinct advantages over hESCs. hiPSC use circumvents the ethical concerns associated with the creation of hESC lines from embryos as they are generated from somatic cells such as blood, urine, and skin (Green, 2019). In terms of personalized medicine, lines can be created from the patient themselves with a wide range of applications including disease modeling *in vitro* to better understand the pathophysiology and provide targets for novel therapeutic development and testing (Doss and Sachinidis, 2019; Ortiz-Vitali and Darabi, 2019). For example, histone deacetylase 4 (HDAC4) was shown to be mislocalized in patient hiPSC-derived dopaminergic neurons modeling Parkinson’s disease, causing downregulation of critical genes (Lang et al., 2019). Treatment of these neurons with compounds that specifically inhibited MAP4K4 action corrected HDAC4 mislocalization and rescued the Parkinson’s disease phenotype (Lang et al., 2019). In patients with a confirmed genetic diagnosis, gene editing could be used correct the mutation in their specific hiPSC line (Yanai et al., 2019); for instance, CRISPR/Cas9 editing of a deep intronic mutation in *CEP290* removed the cryptic splice site and restored CEP290 expression (Burnight et al., 2018). Gene editing can also be used to introduce a known mutation into wild type hiPSCs where patient cells are not available as demonstrated by the generation of an hiPSC line with a single base insertion in the *COL1A1* gene (c.3969_3970insT) found in patients with osteogenesis imperfecta (Hosseini Far et al., 2019). Introducing a known mutation into wild type hiPSCs can also be used as a control in disease models to ascertain its causative nature e.g., assessing the pathogenicity of induced *MYL3* variants associated with hypertrophic cardiomyopathy (Ma et al., 2018b). hESCs can also be engineered to contain a disease-causing mutation for the same *in vitro* disease-modeling as hiPSCs, as demonstrated by the introduction of *CHCHD2* mutations such as c.376C >T, p.(Gln126*) for modeling of Parkinson’s disease and mitochondrial dysfunction (Zhou et al., 2019).

## Sources of Induced Pluripotent Stem Cells and Reprogramming Methods

**Figure 1:**
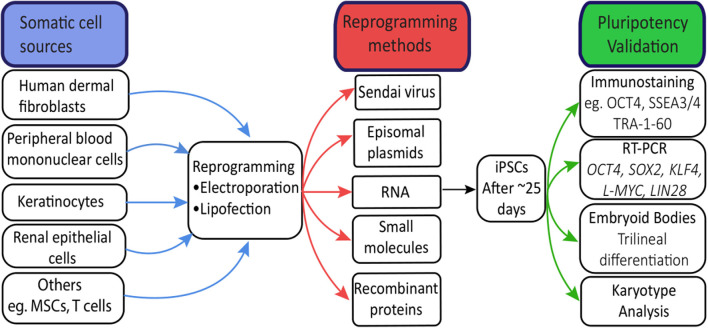
Human-induced pluripotent stem cells (hiPSC) reprogramming. Common sources of somatic cells are reprogrammed by electroporation or lipofection. Induced overexpression of the Yamanaka factors by reprogramming methods drives hiPSC generation, visible after approximately 25 days as tightly packed colonies. Validation of hiPSCs through immunostaining and RT-PCR confirms expression patterns and levels of key pluripotency genes. Trilinear differentiation of embryoid bodies confirms the differentiation capacity of hiPSCs to all three germ layers, while karyotype analysis confirms no chromosomal abnormalities resulting from the reprogramming process.

In principle, hiPSCs can be derived from any somatic cell (Raab et al., 2014). hiPSCs have been most commonly derived from cell sources such as skin, blood, urine, and hair (Takahashi et al., 2007; Wang et al., 2013; Agu et al., 2015; Cheng et al., 2017; Figure 1). Less invasive procedures such as urine collection or blood sampling will encourage more patient donors, particularly children, as this avoids a general anesthetic (Chen et al., 2013). Due to its safety and accessibility, blood is currently the most widely-used source of cells for reprogramming to hiPSCs (Sharma et al., 2018). Reprogramming efficiencies and kinetics vary greatly between each somatic cell type used (Raab et al., 2014; see Supplementary Tables S1, S2 for a comprehensive overview of reprogramming techniques and somatic cell sources).

## hiPSC Modeling of Human Eye Development

### From hiPSCs to Optic Cups

Nakano et al. (2012) developed a protocol for the creation of self-organizing optic cups complete with photoreceptors, retinal neurons and Muller glial cells using hESCs, building on extensive knowledge of retinal differentiation pathways *in vitro* gained through previous experimentation (Meyer et al., 2009, 2011; Nakano et al., 2012). Additionally, *in vivo* studies suggested the coordinated inhibition of critical signaling pathways such as Wnt/BMP and activation of others such as IGF were critical for ocular development (Llonch et al., 2018). It was hypothesized that the modulation of these specific pathways in tightly-controlled culture conditions could generate three-dimensional *in vitro* optic vesicles and mature retinal tissue.

Initially, embryoid bodies (9,000 cells/well) were formed in the presence of Y-27632, a selective inhibitor of Rho-associated coiled-coil containing protein kinase (ROCK) that reduces dissociation-induced apoptosis in hiPSCs and maintained in suspension culture. Embryoid bodies were initially cultured in retinal differentiation media from day 0 to 18. Basal media was supplemented with 20% knock-out serum residue (KOSR) alongside extracellular matrix Matrigel (1%) until day 18. Smoothened agonist (SAG) was added until day 12 to activate the hedgehog signaling pathway and replaced with Wnt agonist CHIR99021 from day 15 to 18. At day 18, differentiating optic cups were transitioned to an NR culture media comprised of DMEM/F12 and N2, a supplement promoting neural differentiation. From day 24, optic vesicle-like structures were excised from larger cell aggregates and retinoic acid, an essential signaling molecule involved in human eye development, was added to culture media to enhance optic cup differentiation. Through temporal control of culture conditions by extrinsic modulation of Wnt, fibroblast growth factor (FGF) and SHH signaling pathways that initially promote eye-field formation in the anterior plate and subsequent eye development, the group successfully generated a protocol modeling the patterning and evagination of the optic vesicle and the invagination of the bilateral optic cup. Both bright-field and confocal microscopy showed striking morphological changes in the first 30 days of differentiation and specification of cellular layers corresponding to early human ocular development.

**Figure 2:**
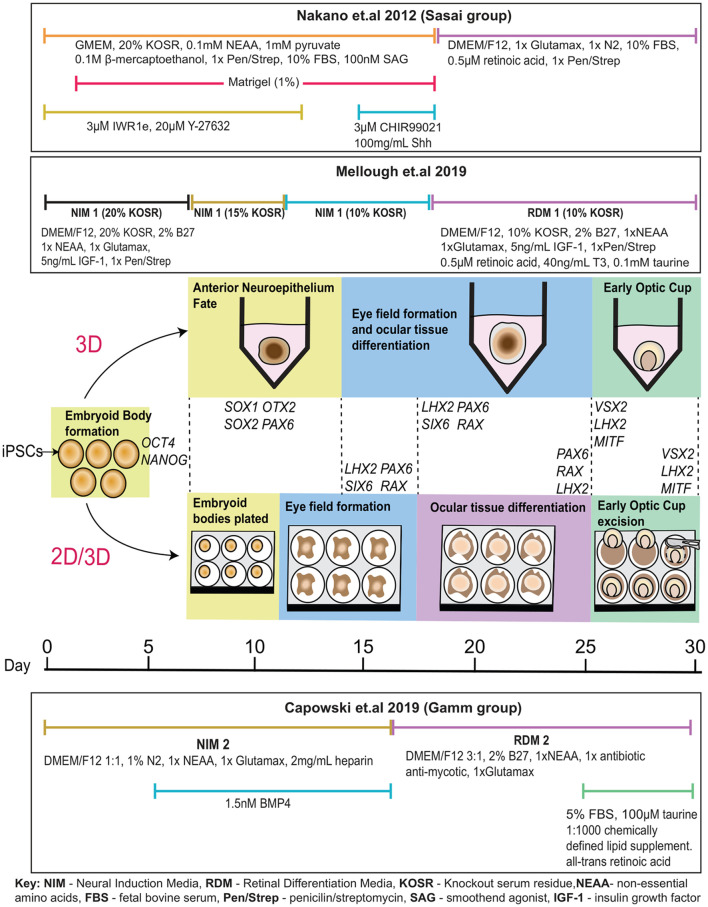
*In vitro* optic cup differentiation with relevant genes expressed at each stage. Following the Sasai protocol, embryoid bodies are formed in the presence of ROCK inhibitor Y-27632 and cultured in a neural induction media supplemented with Wnt inhibitor IWR1e from day 0 to 12, and Wnt and SHH agonists CHIR99021 and Shh from day 15 to 18. Matrigel is added from day 2 to 18. Cells are transitioned to a retinal differentiation media supplemented with N2 and retinoic acid from day 18. According to the Mellough protocol, embryoid bodies are formed in the presence of ROCK inhibitor Y-27632 and cultured in a neural induction media supplemented with IGF-1 and B27 with decreasing knock-out serum residue (KOSR) concentrations adjusted from 20% to 15% at day 7 and from 15% to 10% at day 11. At day 18, cells are transitioned to a retinal differentiation media supplemented with retinoic acid, taurine, and triiodothyronine (T3). Following the 2D/3D differentiation technique most recently described by Capowski et al. (2019); embryoid bodies are formed from iPSCs after 2 days of culture with ROCK inhibitor Y-27632. Cells are weaned into a neural induction media containing N2 and supplemented with BMP4 from day 6 to day 16. At day 7, embryoid bodies are plated to differentiate as a 2D monolayer of cells. The eye field forms around day 10, as cells are guided towards optic cup-like structures. At day 16, cells were transitioned to retinal differentiation media supplemented with 2% B27. By ~25 days, optic cup-like structures are visible and are excised from the adherent culture for further maintenance in suspension and cultured in retinal differentiation media supplemented with FBS, taurine, retinoic acid, and a chemically defined lipid supplement.

Although initial experiments were performed with hESCs rather than hiPSCs, the work of Nakano and colleagues provided huge promise in the modeling of human ocular development and further understanding disease pathophysiology using hiPSCs. Many adaptations of the original protocol have differentiated hiPSCs to a retinal lineage and maintained a completely three-dimensional differentiation system (Figure 2; Kuwahara et al., 2015; Arno et al., 2016; Parfitt et al., 2016; Völkner et al., 2016; Wahlin et al., 2017). Novel three-dimensional protocols have also been developed where cells were differentiated in a descending concentration gradient of KOSR (20% from day 2, 15% from day 7 and 10% from day 11 and onwards) and in the presence of IGF-1 and B27, a supplement promoting growth and viability of central nervous system-associated neurons (Mellough et al., 2015). Remarkably, these protocols recapitulate ocular development despite the absence of *in vivo* cues such as the interaction between the optic vesicle and the surface ectoderm that induces optic cup invagination (Oltean et al., 2016).

Zhong et al. (2014) attempted to induce an anterior neuroepithelial fate in attached cells before directing them to a neuroretina (NR) fate (Zhong et al., 2014). Embryoid bodies were formed in the presence of Blebbistatin rather than ROCK inhibitor Y-27632 and transitioned to a neural induction media containing N2, to promote anterior neuroepithelium formation. Aggregates adhered to culture dishes on day 7 and were cultured in neural induction media containing B27 from day 16, until horseshoe-shaped neuroretinal domains were excised and cultured to form optic cups. Despite the physical constraints of a two-dimensional culture system, optic vesicle, and cup formation were observed (Zhong et al., 2014). This protocol differed by relying on autonomous retinal differentiation guided through *in vitro* intrinsic cues (Zhong et al., 2014; Achberger et al., 2018). Similar results were described by Reichman et al. (2017) who excised optic cups out of culture at day 28 (Reichman et al., 2017), and Gonzalez-Cordero et al. (2017) who excised NR vesicles between weeks 4–7.

Interestingly, the majority of recent protocols have combined two-dimensional and three-dimensional culture, opting to create optic vesicles and cups at an adherent stage before committing cells to long-term three-dimensional differentiation (Figure 2; Zhong et al., 2014; Lowe et al., 2016; Wahlin et al., 2017; Achberger et al., 2018). These protocols are advantageous due to the high level of scrutiny when excising retinal tissue from a monolayer of cells and the reduction of intra- and inter-culture variability (Capowski et al., 2019). Exclusively three-dimensional protocols are advantageous as they closely recapitulate retinal microarchitecture, generate a high percentage of retinal cells, and facilitate self-organization to mature ocular tissue with high fidelity to the human eye development (Capowski et al., 2019; Mellough et al., 2019b). However, these protocols are disadvantageous due to the emergence of ectopic retinal cells, and abnormal structures in culture, loss of inner cell types due to lengthy culture periods and increased variability amongst vesicles.

In most protocols, extrinsic modulation of differentiation cues decreases with time, based on the assumption that long-term differentiation gradually becomes guided by the intrinsic cues found in the differentiating tissue itself (Achberger et al., 2018). Morphological changes form the basis of a recently described rigorous stage-specific selection of optic vesicle-like structures for further differentiation to optic cups (Capowski et al., 2019).

A particular difficulty in optic cup formation lies in the variable efficiency of optic cup invagination *in vitro*, although stratified neuroretina formation is still efficiently induced even in the absence of invaginated optic cups (Nakano et al., 2012; Llonch et al., 2018). Optic cup formation has been reported at efficiencies ranging from 7 to 70% dependent on hiPSC line, reflecting an inherent difficulty in hiPSC modeling (Capowski et al., 2019; Mellough et al., 2019b). Efficiencies can also vary between subsequent differentiation of the same hiPSC line, posing a further difficulty for the generation of hiPSC-derived optic cups (Capowski et al., 2019).

### A Faithful Model of Eye Development

**Figure 3:**
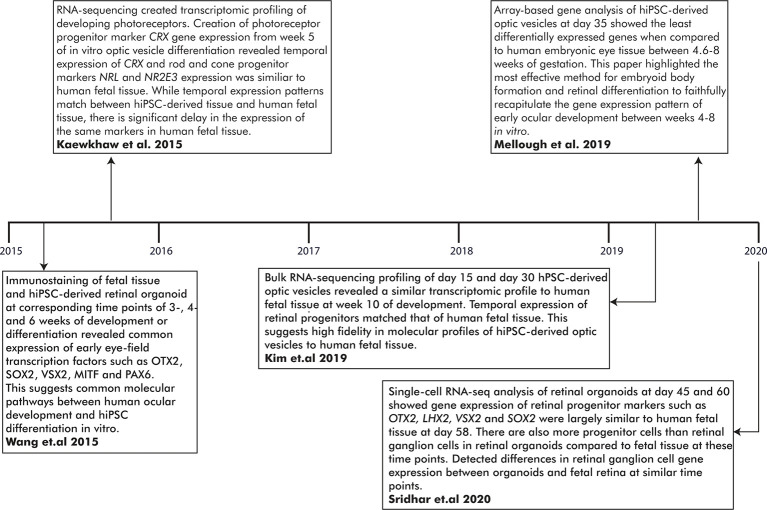
Important milestones in establishing the fidelity of *in vitro*-generated optic vesicles to human fetal tissue (HFT) at early developmental time points. RNA-sequencing has generated transcriptomic profiles of hiPSC-derived optic vesicles and HFT. A comparison of these profiles has revealed novel genes involved in ocular development and highlighted the high molecular fidelity of *in vitro* optic vesicle development to human embryological development.

The fidelity of previously published methods of hiPSC optic cup differentiation was established based on the expression of EFTFs and NR/RPE cell markers at appropriate stages of differentiation. However, comparative analysis with human fetal tissue (HFT) has been limited. Wang et al. (2015) differentiated hiPSCs to optic cups and detected the expression of key EFTFs during early ocular development for comparison with human fetal optic cups (Wang et al., 2015). Similar expression patterns were detected between the two tissue types; for instance, immunostaining revealed PAX6 and OTX2 expression was ubiquitous through both fetal and *in vitro* hiPSC-derived optic vesicles (Wang et al., 2015). Furthermore, MITF and OTX2 co-expression was detected in the RPE layer of both the human fetal and hiPSC-derived optic cups while SOX2 and VSX2 co-expression was observed ubiquitously in the NR layer (Wang et al., 2015). Overall, this study demonstrated consistency between the development of both *in vitro* and fetal bi-layered optic cups using several known stage-specific transcription factor markers (Figure 3).

Transcriptomic profiling of the human fetal ocular tissue has provided a more in-depth molecular insight into the developmental processes underlying ocular development. RNA-seq is an accurate and advanced high-throughput sequencing tool to evaluate temporal and differential gene expression between cell types and/or developmental stages, or delineate genetic networks underlying cell morphology (Mellough and Lako, 2016; Hoshino et al., 2017). The molecular mechanisms of the developing retina have been dissected in several studies yet only one has generated a transcriptomic profile of the early stages of eye development (Young et al., 2013; Aldiri et al., 2017; Hoshino et al., 2017; Welby et al., 2017; Mellough et al., 2019a). Mellough et al. (2019a) reported low expression levels of *PAX6* and *VSX2* despite upregulated *MITF* expression during optic cup formation (Mellough et al., 2019a). *SIX6* and *RAX* were also expressed at low levels although higher than *PAX6* and *VSX2*. *LHX2*, *SOX2*, and *VIM* were highly expressed at the optic cup stage, along with WNT, FGF, and BMP4 pathway genes including *WNT11, FGF19, BMP7*, and *BMP4*. In a later study, comparable *VSX2, OTX2*, and *ASCL1* expression levels were found in optic cups at week 5 compared to the fetal retina at a similar time point, although *FGF5* expression was elevated in the *in vitro* optic cups (Mellough et al., 2019b; Figure 3).

In modeling human ocular development *in vitro*, Kim and colleagues reported molecular congruency between day 15 and day 30 hiPSC-derived ocular tissue and HFT (Kim et al., 2019). Time-course analysis created four clusters of differentially expressed genes associated with different developmental stages of the eye. Differentiating hiPSCs at day 15 yielded highly expressed genes involved in the Wnt and BMP pathways and the developing forebrain including *BMP4, BMP7, WNT1*, and *VAX1* (Slavotinek et al., 2012; Kim et al., 2019). *LHX2* is highly expressed both at day 15 and 30 but significantly downregulated at future time points. By day 30, genes expressed during the optic cup and lens formation such as *VSX2*, or *CRYAA, CRYB4A*, and *CRYBB2* were significantly upregulated compared to day 15; expression levels closely mirrored those detected in HFT (Kim et al., 2019). Also, RNA splicing events and immunostaining expression patterns in differentiating optic cups mirrored those observed in HFT (Kim et al., 2019; Figure 3).

This initial RNA-seq data indicates a high-fidelity hiPSC-derived model of ocular development with extensive cellular and molecular similarities. Further improvement of these models, such as reduction of batch variability, will create more consistent data sets between studies. Additionally, a complete characterization of ocular development requires accessible HFT from earlier developmental stages than optic cup formation (Lindsay et al., 2016). Currently, transcriptomic profiles of the developing human retina do not include data from the early embryological stages of ocular development and cannot be compared with hiPSC models recapitulating those early processes (Aldiri et al., 2017; Hoshino et al., 2017). Future omics studies will provide further insight into early eye development and could be utilized for further understanding of ocular maldevelopment in an hiPSC-derived model at the single-cell level. For a summary of the major accomplishments of omics studies in establishing the molecular fidelity of *in vitro* hiPSC-derived optic vesicles to early human eye development, see Figure 3.

## Developmental Eye Disorders and Associated Genetic Variants Modelled Using Hipscs

### Early Eye Development

#### Morphogenesis and Gene Regulatory Networks

**Figure 4:**
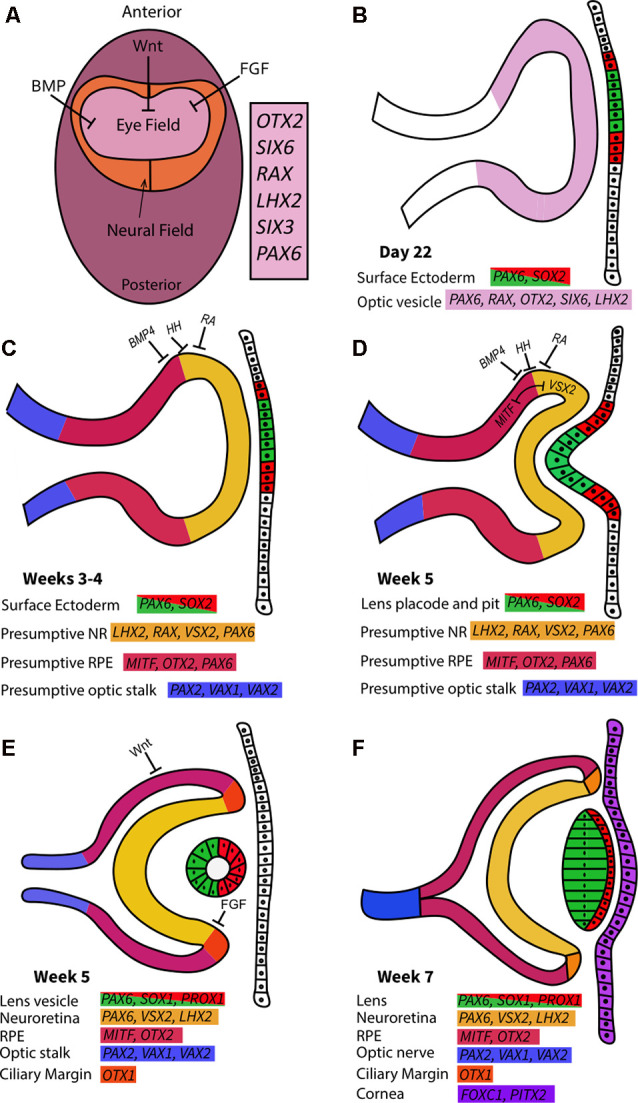
Early ocular morphogenesis. **(A)** Developmental pathways such as Wnt, BMP, and fibroblast growth factor (FGF) drive upregulation of eye-field transcription factors in the anterior neural plate, creating the specified region known as the “eye-field.” **(B)** Deepening of optic sulci and evagination of optic vesicle around 22 days post-conception. The newly formed optic vesicle ubiquitously expresses all eye-field transcription factors. **(C)** The action of signaling pathways determines presumptive regions in the optic vesicle characterized by unique gene expression patterns in the third and 4th weeks of gestation. **(D)** Interactions between the optic vesicle, surface ectoderm, and extraocular mesenchyme cause the invagination of the optic cup at approximately 5 weeks post-conception. MITF and VSX2 interactions create boundaries between retinal pigment epithelium (RPE) and neuroretina (NR) in the developing optic cups. The lens pit begins to form from the surface ectoderm. **(E)** In the 5th week of gestation following optic cup formation, Wnt and FGF pathways drive RPE/NR differentiation and clear definition of these regions through ciliary margin formation. The lens vesicle forms as the lens pit detach from the surface ectoderm. **(F)** By the 7th week of gestation, lens fibers extend to form the lens from the hollow lens vesicle. The cornea forms from the overlying surface ectoderm. NR and RPE are clearly defined and separated by the ciliary margins, while the optic nerve forms from the convergence of the optic stalk.

Vertebrate eye development is tightly controlled by spatiotemporal gene expression patterns and interactions between the embryonic germ layers (Figure 4; Harding and Moosajee, 2019). The eye is derived from: (i) neuroectoderm, which gives rise to the neural retina (NR), retinal pigment epithelium (RPE), optic nerve, iris dilator and sphincter muscles, and ciliary body; (ii) surface ectoderm, which contributes to the lens, conjunctival and corneal epithelia; and (iii) mesenchyme, which originates from the mesoderm and neural crest cells, forming the corneal endothelium and stroma, iris stroma, ciliary muscles, vasculature, and sclera. Human eye development is first evident at around day 22 of gestation and is not completed until several months after birth.

Following gastrulation and specification of the three germ layers, the formation of the eye field from the anterior neural plate takes place, this is characterized by expression domains of early eye-field transcription factors (EFTFs) *PAX6, OTX2, RAX, SIX6, SIX3* and *LHX2* (Figure 4A; Chen et al., 2017). The complex interactions between various signaling cascades and EFTFs are crucial to produce the appropriate cell types at the correct developmental time and ensure their correct optimal cell proliferation, migration, and polarity (Gregory-Evans et al., 2013). EFTFs regulate signaling pathways intrinsic to ocular tissue that drives the morphogenetic events of ocular development, such as optic vesicle and cup formation. Mutations in the genes encoding EFTFs lead to ocular maldevelopment.

**Figure 5:**
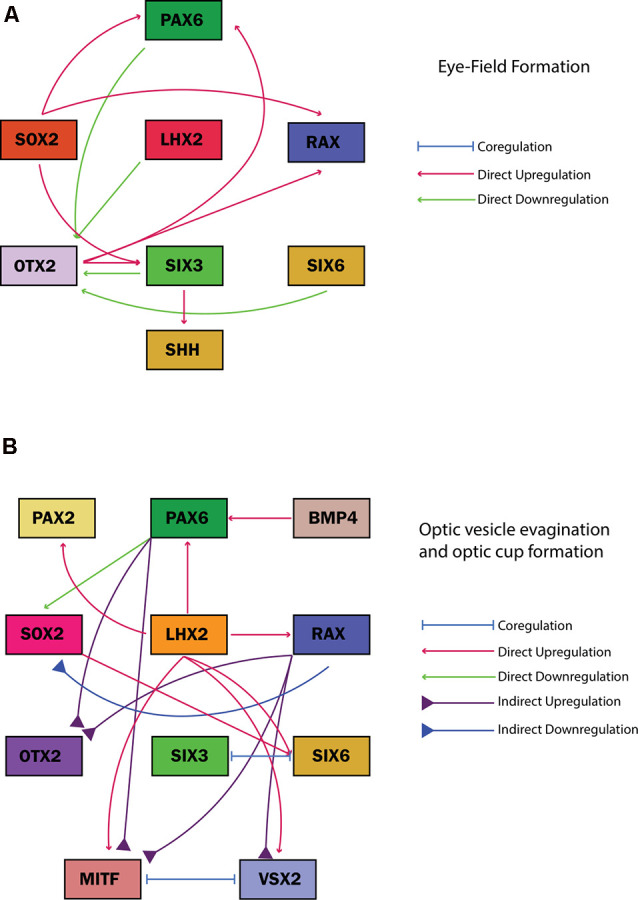
Gene regulatory networks of common eye field transcription factors associated with ocular malformation guiding cell fates during **(A)** eye field formation and **(B)** optic vesicle evagination and optic cup formation. The regulatory effect of each transcription factor on the other is illustrated in the key adjacent to each figure along with the specific developmental stage.

*OTX2* is initially expressed in the anterior neuroepithelium and is the earliest molecular marker in the eye field, together with *SOX2*, it activates *PAX6, RAX*, and *SIX3* expression and is subsequently downregulated (Figure 5A; Danno et al., 2008; FitzPatrick, 2016; Giger and Houart, 2018). *SIX3* regulates *SHH* expression in the sonic hedgehog (SHH) signaling pathway, required for dorso-ventral patterning of the forebrain and later modeling of the optic vesicle and cup, through canonical Wnt pathway antagonization (Jeong et al., 2008; Diacou et al., 2018). *RAX*, an early marker of ocular development, is critical for retinal progenitor proliferation and optic vesicle evagination but later restricts *OTX2* expression in the eye field (Figure 5A; Gregory-Evans et al., 2013; Zagozewski et al., 2014; Rodgers et al., 2018). *RAX* variants are associated with anophthalmia and microphthalmia (Reis and Semina, 2015). *SOX2* and *OTX2* expression in the early eye field regulate *RAX* by binding to a conserved enhancer element containing binding sequences for both factors (Danno et al., 2008; Slavotinek, 2019). *TBX3* is also expressed in the eye-field and induces neural induction and normal eye formation by repressing *BMP4* expression, and maintaining these eye-field neural progenitors in a multipotent state before retinal induction (Motahari et al., 2016).

The first morphological milestone in eye development is the bilateral evagination of the eye field region, which occurs during neurulation. The splitting of the eye field first appears as small indentations known as optic sulci at day 22 post-conception (Figure 4B; Harding and Moosajee, 2019). SHH and FGF signaling pathways, regulated by transforming growth factor (TGF) signaling, initiate the splitting of the eye field and the subsequent posterior to anterior migration of cells to drive optic vesicle evagination (Cardozo et al., 2020). SHH signaling originating from the midline tissue in the ventral forebrain by regulating the inverse expression of *PAX2*, expressed in the presumptive optic stalk, and *PAX6*, expressed in the presumptive neural retina and pigment epithelium, to mediate the partitioning of the optic primordia to optic stalk and presumptive optic cup (Macdonald et al., 1995).

Eph/Ephrin signaling likely plays a role mediating Wnt, SHH, and FGF signaling to control optic vesicle evagination as it branches off from the anterior forebrain (Cardozo et al., 2020). Failure of the eye field to split results in a single central eye known as cyclopia, often caused by defects in dorso-ventral patterning associated with *SHH* mutations (FitzPatrick, 2016; Placzek and Briscoe, 2018). LHX2 is required for correct optic sulci localization and loss of expression after optic vesicle formation may arrest optic cup formation (Roy et al., 2013). However, *LHX2* variants associated with anophthalmia have only been described in a mouse model and have not been linked to any ocular malformations in humans (Desmaison et al., 2010; Plaisancié et al., 2019).

The optic vesicles form as the optic sulci deepen in the 4th week of embryonic development. They are connected to the developing forebrain by the optic stalk which later develops into the optic nerve. Early EFTFs such as *PAX6* and *OTX2* are expressed in the optic vesicle and are required for cell fate-determining signaling pathways (Fuhrmann, 2010). Co-expression of *SOX2* and *PAX6* is ubiquitous throughout the optic vesicles (Hever et al., 2006; Matsushima et al., 2011; Kondoh et al., 2016). *RAX* expression is upregulated by LHX2 in the eye field and the evaginating optic vesicle (Figure 5B; Gregory-Evans et al., 2013).

Intrinsic and extrinsic factors provide patterning cues to establish molecular boundaries in the optic vesicles, while a distinct set of transcription factors are expressed in presumptive regions of the optic vesicle for the development of each cell type (Figure 4C; Heavner and Pevny, 2012; Giger and Houart, 2018). SHH, retinoic acid, and BMP4 signaling pathways create unique molecular regions, such as the *PAX6*-expression domain specified by BMP4 modulation in the distal optic vesicle (Figure 4C; FitzPatrick, 2016). Disruptions to this process can arrest eye development; for example, mutations in BMP4 antagonist *SMOC1* result in anophthalmia (Rainger et al., 2011).

Towards the end of the 4th week of gestation, the distal optic vesicle contacts the overlying surface ectoderm allowing BMP and retinoic acid released from the lens placode to bind to the optic vesicle and displace the intervening mesenchyme (Snell and Lemp, 2013; Harding and Moosajee, 2019). Within this region of contact, each optic vesicle and surface ectoderm thickens to form placodes and invaginate to form the optic cup and lens pit respectively (Figure 4D). The invagination of the optic vesicle to form the bi-layered optic cup is stimulated by BMP4 and retinoic acid (Harding and Moosajee, 2019). The inner layer develops into the NR, while the RPE is formed from the external layer. *TBX3* activates Noggin induction of *PAX6* expression and co-expression with *TBX3* drives retinal differentiation in the eye field (Motahari et al., 2016). *SOX2* expression is downregulated in the presumptive RPE region, upregulating *MITF* and *OTX2* expression to drive RPE formation (Figure 5B; Kondoh et al., 2016; Chen et al., 2017). *SOX2* and *PAX6* are expressed at an inverse gradient in the invaginating optic cup where *SOX2* has a critical role in maintaining the potential for neuronal differentiation as its expression is gradually confined to the outer layer of the NR (Chen et al., 2017). Loss of SOX2 reduces the capacity for NR formation during optic cup invagination and causes preferential differentiation to the non-neurogenic ciliary epithelium (Matsushima et al., 2011). Optic cup malformation caused by loss of SOX2 function is possibly due to the failed antagonism of the Wnt/β-catenin pathway that results in impaired NR/RPE differentiation and optic cup formation (Kelberman et al., 2008; Capowski et al., 2016). Accordingly, a large proportion of *SOX2* loss-of-function mutations cause anophthalmia (Slavotinek, 2019).

The reciprocal relationship between *MITF* and *VSX2*, coupled with FGF signaling, drives the establishment of pronounced NR and RPE domains (Figure 4E; Capowski et al., 2014). *LHX2* also acts upstream of *MITF, VSX2*, and *PAX2* for temporal control of their expression (Figure 5B; Chou and Tole, 2019). β-catenin/Wnt signaling specifies the RPE fate in the dorsal optic cup by directly upregulating *MITF* and *OTX2* expression while FGF signaling acts primarily through FGF9 through promoting the development of the ciliary margin at the NR/RPE junction (Figure 4E; Westenskow et al., 2009; Balasubramanian et al., 2018).

The proximal portion of the newly-formed optic vesicle expresses *PAX2, VAX1* and *VAX2* genes that are responsible for optic stalk formation (Figure 4D; Stanke et al., 2010; Patel and Sowden, 2019). In the evaginating optic vesicle, LHX2-regulated BMP4 expression induces the formation of the lens placode in the overlying surface ectoderm while PAX6 and SOX2 simultaneously bind to enhancers in the lens placode to drive early lens crystallin production, targeting critical genes such as *FOXE3* (Harding and Moosajee, 2019). The deceleration of cell division at the center of the lens placode causes its invagination, producing lens pits that detach from the overlying surface ectoderm to form the lens vesicle (Plaisancié et al., 2019). After detachment, PROX1 regulates lens differentiation and fiber elongation as *PAX6* expression is maintained in the lens vesicle (Cvekl and Zhang, 2017).

During the 5th week of ocular development, the optic fissure develops as a furrow along the ventral surface of the optic cup extending to the optic stalk (Plaisancié et al., 2019). This transient structure enables the vasculature to enter and supply the developing eye and completely fuses around week 7 (FitzPatrick, 2016; Richardson et al., 2017). Ocular coloboma will result from incomplete optic fissure fusion and has been associated with various genetic variants, including *PAX6* and *PAX2* that cause disrupted optic nerve/RPE boundaries (ALSomiry et al., 2019). Following optic fissure fusion, the formation of the major eye structures is mostly complete and maturation of the ocular tissues occurs subsequently.

By approximately the 6th week of gestation, interactions between the lens and optic cup induce the formation of the cornea from the surface ectoderm (Davies et al., 2009; Snell and Lemp, 2013). During the 7th week of embryonic development, periocular mesenchymal and neural crest cells migrate into the space between the surface ectoderm and the lens vesicle in three distinct waves to gradually form the corneal stroma, epithelium and endothelium (Figure 4F; Lwigale, 2015). The third wave of mesenchymal cell migration also contributes to iris formation (Eghrari et al., 2015). *PITX2* and *FOXC1* are key transcription factors expressed in the periocular mesenchyme regulating corneal development; mutations in these genes are associated with anterior segment dysgenesis (Hara et al., 2019). *PAX6* expression is still critical at this point for the development of the anterior segment structures originating from the mesenchyme (Cvekl and Tamm, 2004; see Supplementary Table S3 for a comprehensive overview of the genes associated with development eye disorders).

### Microphthalmia

Microphthalmia is defined by the presence of an eye with an axial length of more than two standard deviations (SD) below the age-adjusted population mean (21 mm in adults and <14 mm in newborns; Harding and Moosajee, 2019). Severe microphthalmia is characterized by a corneal diameter of <4 mm associated with a total axial length <10 mm at birth or <12 mm after 1 year (Harding and Moosajee, 2019). In complex cases, microphthalmia can be associated with other anterior or posterior segment abnormalities (Plaisancié et al., 2019). The common causative genes encode EFTFs such as *SOX2, PAX6, VSX2*, and *OTX2*, or those that encode components of the retinoic acid signaling pathway such as *STRA6* or *ALDH1A3* (Williamson and FitzPatrick, 2014). Chromosomal abnormalities are responsible for approximately 7–15% of syndromic cases (Eintracht et al., 2020).

To investigate ocular maldevelopment, hiPSCs containing a homozygous *VSX2* mutation (p.Arg200Gln) associated with microphthalmia were differentiated to early optic cups (Joseph Phillips et al., 2014). The early stages of ocular development were not impaired by the *VSX2* mutation, demonstrated by consistent eye field marker expression and the appearance of morphologically indistinguishable VSX2*+* proliferative optic vesicle-like structures in both the wild type and mutant cultures. However, a disease-like phenotype was apparent after optic vesicle formation as mutant vesicles did not proliferate at the same rate as the healthy control. Mutant optic vesicles also showed a greater preference towards an RPE than NR fate, disrupting optic cup formation. This observation was verified using comparative RNA-seq analysis, where genes involved in Wnt and TGF signaling that drive RPE differentiation, such as *WNT11* and *BMP8A* respectively, were upregulated in mutant vesicles during optic cup formation. Simultaneously, genes involved in FGF signaling driving NR formation, such as *FGF19*, were downregulated in mutant vesicles.

Further investigation into the molecular etiology of microphthalmia, using the same patient hiPSC line, highlighted the transcriptional regulatory role of VSX2 (Capowski et al., 2016). In wild type vesicles, VSX2 and MITF were expressed exclusively in NR and RPE progenitor cells respectively by day 18 yet VSX2 and MITF co-expression was detected in *VSX2*-mutant vesicles. β-catenin, a marker of canonical Wnt pathway activation, was also co-expressed with VSX2+ cells in mutant but not wild type vesicles, indicative of dysregulated Wnt signaling. Altogether, the results suggested that defective VSX2 binding activity fails to repress Wnt signaling and MITF expression that is required to induce correct NR/RPE differentiation and optic cup formation. Tightly-controlled pharmacological inhibition of the Wnt pathway during specific windows of optic vesicle development in *VSX2* mutant vesicles restored functional NR differentiation and disrupted VSX2/MITF colocalization in the developing optic cup. VSX2+ cells were detected in a similar abundance and pattern as wild type vesicles, although MITF expression was not detected in treated vesicles. This partially rescued the disease phenotype as the NR layer of the bi-layered optic cup was restored, but not the RPE layer. Pharmacological activation of the Wnt pathway in wild type vesicles also effectively recapitulated the phenotype observed in *VSX2* mutant vesicles.

A recent study revealed that *FGF9* and *FGF19* were expressed at different times in the optic vesicles to direct early NR development and their expression was found to be reduced in the *VSX2* mutant (Gamm et al., 2019). NR differentiation was enhanced by FGF9 supplementation, as ERK1/2 expression was transiently upregulated and partially rescued the mutant phenotype observed in the *VSX2*-mutant vesicles. ERK1/2 is part of the ERK/MAP pathway that promotes cell proliferation (Mebratu and Tesfaigzi, 2009). The reverse action of withholding FGF9 did not promote a non-NR fate. This possibly indicates a role for VSX2 in concert with FGF9 to promote NR development (Gamm et al., 2019). Despite limited work, the use of patient hiPSC-derived optic vesicles has already provided some insight into the pathophysiology of *VSX2*-related microphthalmia and highlighted potential therapeutic targets.

Modeling of microphthalmia-associated with the *VSX2* mutation (p. Arg200Gln) was only performed with one patient-specific hiPSC line and one unaffected sibling control (Joseph Phillips et al., 2014; Capowski et al., 2016; Gamm et al., 2019). Consequently, the reliability or reproducibility of published results can be questioned and larger sample size is required. These studies may skew our understanding of disease phenotype, etiology, and pathophysiology. The choice of age-, sex- and ethnicity-matched controls is an important consideration to minimize variability between cell lines (Ortmann and Vallier, 2017; Takasaki et al., 2018; Victor et al., 2018; Deneault et al., 2019).

Introducing specific mutations in wild type iPSC lines through CRISPR/Cas9 gene editing can create isogenic disease models. A comparison of isogenic and patient-derived disease models can ascertain the causative nature of disease-associated variants, such as the *VSX2* mutation (p. Arg200Gln) associated with microphthalmia. As an isogenic iPSC-derived disease model of *VSX2*-associated microphthalmia has not been produced alongside these patient models, it is harder to predict the causative nature of the *VSX2* mutation.

### Corneal Hereditary Endothelial Dystrophy

Corneal hereditary endothelial dystrophy (CHED; OMIM:217700) is a rare autosomal recessive disorder leading to severe visual impairment caused by bilateral corneal edema characteristic of primary endothelial cell dysfunction (Brejchova et al., 2019). A subset of patients also suffers from progressive sensorineural hearing loss, in a condition known as Harboyan syndrome (Desir and Abramowicz, 2008; Brejchova et al., 2019). CHED is associated with biallelic pathogenic *SLC4A11* variants, where loss-of-function mutations cause cell adhesion and ion transport defects that reduce corneal endothelial cell viability (Brejchova et al., 2019; Malhotra et al., 2020). This developmental defect occurs around the 7th week of gestation as migrating periocular mesenchymal and neural crest cells begin to differentiate into specialized corneal cell types (Lwigale, 2015; Brejchova et al., 2019).

To characterize the effects of *SLC4A11* variants on corneal endothelial (CE) cells, Brejchova et al. (2019) differentiated both healthy and six patient-hiPSC lines with an *SLC4A11* mutation (c.2240 + 5G >A) to CE cells (Brejchova et al., 2019). Detection of corneal markers ZO-1, N-Cadherin, and CD166 confirmed the identity of differentiated cells. Eleven pathogenic variants were identified, including an alternatively spliced transcript detected in one patient line, revealing a cryptic donor site introduced by the c.2240 + 5G >A mutation. This led to the insertion of six bases, resulting in a premature stop codon (p. [Thr747*]).

One patient, compound heterozygous for c.2240 + 5G >A, p.(Thr747*) and c.625C >T, p.(Arg209Trp), in this study had late-onset CHED compared to the congenital form observed in all other patients. This variant was not located within the canonical splice site and its pathogenicity could not be determined without an experimental model. As *SLC4A11* is only expressed in CE cells, these needed to be generated from hiPSCs due to the inaccessibility of appropriate HFT.

Changes to *SLC4A11* splicing detected in hiPSC-derived models may inform our understanding of variable disease phenotypes caused by changing quantities of mutant *SLC4A11* transcript due to both alternative splicing and overriding mechanisms. The hiPSC-derived CHED model can be further utilized to investigate changes to SLC4A11 transport function, protein stability, and localization associated with *SLC4A11* variants.

## Limitations of hiPSC Use

Genetic variation imparts a donor-specific genomic and epigenetic signature on hiPSCs that can influence their differentiation capacity and downstream functionality (Kim et al., 2010; Vaskova et al., 2013; Noguchi et al., 2018). Each hiPSC line will have variable differentiation capacities and many cell lines may need to be initially differentiated in pilot experiments to select optimal lines for further experiments (Kyttälä et al., 2016; Cowan et al., 2019). Isogenic hiPSC lines created by gene editing can avoid the genetic variability of multiple hiPSC lines yet effectively model diseased or healthy phenotype (Chakrabarty et al., 2018). Reprogramming efficiency can also be impacted by specific epigenetic enzymes such as the SWI/SNF complex, the age and ancestral origins of the donor (Mackey et al., 2018). The practicalities of hiPSC production also need to be considered. The process to generate hiPSCs is time-consuming and costly, and reprogramming requires specialized equipment and expertise (Giacalone et al., 2016). Additionally, uniquely formulated costly media and reagents handled with a meticulous aseptic technique are required for reprogramming and culture (Giacalone et al., 2016). As there is no consensus on the optimal somatic cell source for reprogramming, it is important to consider the specific advantages and disadvantages of each cell type during experimental design (Supplementary Tables S1, S2, Foltz and Clegg, 2019).

An important disadvantage to consider is the loss of X-chromosome inactivation, the transcriptional silencing of one of the two X-chromosomes in female cells (Geens and Chuva De Sousa Lopes, 2017). This epigenetic regulation of X-chromosomal gene expression is critical to correctly modulating X-chromosome dosage to ensure healthy development. Aberrant X-chromosome states associated with the disease have been detected in hiPSCs and may impact their capability to model development and disease *in vitro* (Geens and Chuva De Sousa Lopes, 2017).

Following iPSC generation, it is critical to ensure any reprogramming vectors or plasmids are completely absent from cells, as common methods such as Sendai or episomal reprogramming can leave a transient footprint on the host genome. To ensure differentiation is not impaired by these vectors or plasmids, low passage (<10) iPSCs should not be used.

hiPSC modeling is inherently limited as it does not replicate the entire biological system with intrinsic and extrinsic cues that control tissue development *in vivo* (Bartfeld and Clevers, 2017). Normal oxygen conditions do not mimic the hypoxic embryological microenvironment of optic vesicle and cup development (DiStefano et al., 2018). Additionally, secondary structures of the developing eye such as vascularization are lacking (Achberger et al., 2018).

It is also important to note that hiPSCs are not hESCs, for whom most of these limitations are not relevant. Yet hESCs have their specific shortcomings such as unclear genotype-phenotype correlations in hESC-based disease models and the need to genetically manipulate hESCs for disease modeling (Halevy and Urbach, 2014). The differences and similarities of hiPSCs and hESCs have been extensively discussed in the literature (Cherry and Daley, 2013; Halevy and Urbach, 2014; Marei et al., 2017; Zhao et al., 2017).

## Future Work

Retina-on-a-chip and retinal differentiation using bioreactors are attempting to enhance *in vitro* differentiation to more closely recapitulate the human embryological environment, such as the outer blood-retina barrier (DiStefano et al., 2018; Achberger et al., 2019). A recently reported retina-on-a-chip system incorporated a vascularized retinal organoid-RPE unit with demonstrated capabilities for drug testing and greater recapitulation of native retina physiology (Achberger et al., 2019). Researchers at the National Eye Institute, USA are developing a three-dimensional *in vitro* RPE/choroid to improve understanding of the photoreceptor/RPE/choroid complex using patient-derived endothelial cells, choroidal fibroblasts and pericytes encapsulated in a collagen-based gel and bio-printed on one side of a biodegradable scaffold with an RPE monolayer derived from the same hiPSCs grown on the other side.

Of the numerous developmental eye diseases that can affect patients, only hiPSC-based modeling of microphthalmia and CHED has been described so far (Joseph Phillips et al., 2014; Brejchova et al., 2019; Gamm et al., 2019). The utility of hiPSC modeling for genetically heterogenous early-onset developmental eye disorders will enhance our understanding of their molecular and epigenetic mechanisms (Gregory-Evans et al., 2019). Future modeling of developmental eye disorders can assess the long-term effects of a mutation in, for example, an EFTF gene through retinal organoids. *VSX2* is involved with bipolar cell differentiation (Clark et al., 2008; Zou and Levine, 2012), and modeling of retinal development in *VSX2*-mutant hiPSCs revealed a complete absence of this cell type, indicating impaired retinal differentiation (Joseph Phillips et al., 2014). These models can provide information on cell patterning to be used for patient phenotyping with advanced ocular imaging techniques and help to predict the response to potential therapies (Ma et al., 2017).

hiPSC-derived models of ocular disease are powerful tools for the development of new therapies and are more physiologically relevant than animal models for pre-clinical testing. Gene editing and antisense oligonucleotide therapy have restored a healthy phenotype from patient-derived retinal organoids with *CEP290* and *RPGR* mutations (Parfitt et al., 2016; Deng et al., 2018). Nonsense suppression using read-through drugs such as PTC124 (ataluren) is a further promising approach which has been reported to restore full-length protein and functionality in *RP2^R120X^*, *MERTK-*deficient and *KCNJ13^W53X^* hiPSC-derived RPE (Schwarz et al., 2015; Ramsden et al., 2017; Shahi et al., 2019). Once hiPSC-derived organoids that effectively recapitulate the hallmarks of each disease have been established, these models will provide ideal pre-clinical platforms for the discovery, testing and development of translational therapeutics.

## Conclusion

Ocular maldevelopment accounts for a third of congenital blindness worldwide, and a genetic component is responsible for the majority of cases. hiPSC modeling of early eye development has advanced greatly in recent years and omics studies reveal a close cellular and molecular similarity with HFT of similar development stage. Improvement of hiPSC modeling protocols will enhance the fidelity of these models to early ocular morphogenesis. The use of hiPSCs to model developmental eye diseases has been effectively demonstrated in a patient-derived model of microphthalmia and CHED. These findings are encouraging for further investigation of many other developmental eye disorders, which will be essential to understand the mechanisms of ocular malformation due to their genetic heterogeneity. This will advance therapeutics testing and inform genetic counseling, to improve the quality of life of both patients and their families affected.

## Author Contributions

JE: writing and original draft. JE, MT, and MM: writing, review and editing. MM: funding.

## Conflict of Interest

The authors declare that the research was conducted in the absence of any commercial or financial relationships that could be construed as a potential conflict of interest.
