# Supplementary material for: The Use of Induced Pluripotent Stem Cells as a Model for Developmental Eye Disorders
Source: Front Cell Neurosci. 2020 Aug 20;14:265. doi: 10.3389/fncel.2020.00265 (PMC7468397; doi:10.3389/fncel.2020.00265)
Supplement: Supplementary file 1 [file Table_1.DOCX]

| **Cell Source** | **Advantages** | **Disadvantages** |
| --- | --- | --- |
| Human dermal fibroblasts | - Stable cell source reprogrammable after 5-10 passages - Cost-effective - Simple to culture | - Lower reprogramming efficiency than other common cell types using same reprogramming methods - Derived through invasive slow biopsy requiring local anesthetic for older children and adults |
| Keratinocytes | - Harvested from hair follicles in non-invasive procedure - Higher reprogramming efficiencies | - Isolation of keratinocytes from hair follicles requires specific expertise - Expensive and difficult to culture as low-calcium media is required to prevent senescence |
| Renal epithelial cells | - Harvest cells from urine sample - Cells can be cultured following freeze/thaw cycles - Epithelial to mesenchymal transition could be the priming critical step for somatic cell reprogramming | - Reprogramming only successful with low passage cultures (<5) - Only ~0.1% of harvested cells adhere in culture due to heterogenous cellular composition of urine |
| Peripheral blood mononuclear cells (PBMCs) | - Less invasive procedure for cell derivation - Large quantities of cells can be harvested from a single patient venous blood sample, so less invasive - Isolated PBMCs do not require same length of time for expansion prior to reprogramming | - Lower reprogramming efficiency than other common cell types using same reprogramming methods |

**Table 1: Overview of commonly used cell sources for hiPSC reprogramming**
