# Supplementary material for: The Use of Induced Pluripotent Stem Cells as a Model for Developmental Eye Disorders
Source: Front Cell Neurosci. 2020 Aug 20;14:265. doi: 10.3389/fncel.2020.00265 (PMC7468397; doi:10.3389/fncel.2020.00265)
Supplement: Supplementary file 2 [file Table_2.DOCX]

| **Reprogramming Method** | **Advantages** | **Disadvantages** |
| --- | --- | --- |
| Episomal plasmid | - Non-integrating plasmid-based method operating through episomal vectors that are maintained extrachromosomally in the cell - Plasmids efficiently removed from cells post-transfection | - Lower reprogramming efficiency |
| Sendai virus | - Does not go through a DNA phase nor integrate into the host genome - Higher reprogramming efficiency - Can transduce most cell types - High protein expression 24hr post-transduction which enhances self-replication - Deletion on envelop-related genes attenuates elicited immune response | - Laborious to produce - Commercially very expensive - Potentially cytotoxic - Prolonged presence in cells post-transduction |
| mRNA | - Non-integrating - Base modification and 5’-capping of synthetic mRNA has reduced immunogenicity, increased stability and improved efficiency - Continually advancing as it is produced synthetically and can be delivered in combination with enhancing factors | - Sensitive to nucleases and heat - Difficult to produce in large quantities - Expensive to produce - Multiple transfections required |
| Small molecule | - Non-integrating - Transgene-free | - Low reprogramming efficiency |
| Recombinant Proteins | - Non-integrating - No involvement from exogenous nucleic acids | - Very low reprogramming efficiency - Lack eukaryotic post-translational modifications |
| Associated Adenovirus vectors (AAVs) | - Split and fragment AAV vectors aim to overcome random integration events by encapsidation of delivered genes or delivery of multiple gene components that are reconstituted *in vitro* | - Recombinant AAVs associated with random integration events - Require multiple transfections |
| Lentivirus | - Higher reprogramming efficiencies | - Integrating viral vector with unpredictable effects on functional integrity or regulation of neighboring genes - Transgene incompletely silenced and may reactivate during differentiation |

**Table 2: An overview of commonly used reprogramming methods**
