# Supplementary material for: The Use of Induced Pluripotent Stem Cells as a Model for Developmental Eye Disorders
Source: Front Cell Neurosci. 2020 Aug 20;14:265. doi: 10.3389/fncel.2020.00265 (PMC7468397; doi:10.3389/fncel.2020.00265)
Supplement: Supplementary file 3 [file Table_3.docx]

| **Eye Disorder** | **Developmental Defect** | **Phenotype** | **Week(s) of gestation when defect occurs** | **Key associated genes (syndromic and non-syndromic forms)** |
| --- | --- | --- | --- | --- |
| Anophthalmia | Absence of optic vesicle, defective optic vesicle patterning and growth, or defective optic cup invagination | Complete absence of eye | Week 4-5 | *ALDH1A3, BCOR, BMP4, BMP7, CSPP1, FNBP4, FRAS1, GDF6, GLI2, HCCS, MAB21L2, MITF, NAA10, OTX2, PAX6, PORCN, RARB, RAX, RBP4, SIX6, SMCHD1, SMOC1, SOX2, STRA6, TBC1D32, TCTN2, TFAP2A, VAX1, VSX2, YAP1* |
| Microphthalmia | Defective optic vesicle patterning and growth. Lack of cell proliferation in optic vesicles | Small underdeveloped eye | Week 4-5 | *ACTB, ACTG1, ADAMTS18, ALX1, ALX3, ATOH7, B3GALNT2, BCOR, BMP4, CPLANE1, C12ORF57, CHD7, COL4A1, CRYBA4, CRPPA, DAG1, DPYD, ERCC1, ERCC5, ERCC6, ESCO2, FAM111A, FANCA, FANCD2, FANCE, FANCI, FANCL, FAT1, FKRP, FKTN, FNBP4, FOXE3, FOXL2, FRAS1, FREM1, FREM2, GDF3, GDF6, GJA1, GLI2, GRIP1, HCCS, HDAC6, HMGB3, HMX1, IKBKG, KATNIP, KIF11, KDM6A, KMT2D, LRP5, MAB21L2, MAF, MAPRE2, MCOLN1, MITF, MKS1, NAA10, NDP, NHS, OLFM2, OTX2, PAX2, PAX6, PDE6D, PITX3, POMGNT1, POMGNT2, POMT1, POMT2, PORCN, PQBP1, PTCH1, PRSS56, PXDN, RAB3GAP1, RAB3GAP2, RAB18, RARB, RBP4, RERE, RIPK4, RPGRIP1L, RXYLT1, SALL1, SALL4, SCLT1, SEMA3E, SHH, SIX3, SIX6, SLC38A8, SMCHD1, SMG9, SMOC1, SMO, SNX3, SOX2, SRD5A3, STRA6, TBC1D20, TBC1D32, TENM3, TFAP2A, TMEM216, TMEM67, TMX3, TUBB, VAX1, WNT3, ZEB2* |
| Coloboma | Incomplete optic fissure closure | Choroid, retina, iris, optic nerve | Week 5-7 | *ABCB6, ACTB, ACTG1, BMP7, C12ORF57, CHD7, CLDN19*, COL27A1*, DHX38, FADD, FOXE3, FZD5, GDF3, GDF6, GJA8, IGBP1*, KIF7, KIAA0556, MAB21L2, MITF, PAX2, PIGL, POLR1C, POLR1D, RARB, RBP4, SALL2, SALL4, SHH, SIX3, SMCHD1, STRA6, TCOF1, TFAP2A, TSPAN12, VPS35L*, VSX2, WDR11, WDR37, WFS1, YAP1, ZEB2* |
| Congenital Cataracts and/or Primary Aphakia | Breakdown of lens microarchitecture and/or absence of lens due to failure of lens induction from surface ectoderm | Opacity of crystalline lens and/or absence of lens | Week 4-7 | *ABHD12, ADAMTS10, ADAMTSL4, AGK, AGPS, ALDH18A1, BCOR, BEST1, BFSP1, BFSP2, CBS, CHMP4B, COL18A1, CRYAA, CRYAB, CRYBA1, CRYBA4, CRYBB1, CRYBB2, CRYBB3, CRYGB, CRYGC, CRYGD, CRYGS, CTDP1, CYP27A1, CYP51A1, DHCR7, DPYD, EPG5, EPHA2, ERCC2, ERCC3, ERCC5, ERCC6, ERCC8, FAM111A,FAM126A, FBN1, FOXE3, FTL, FYCO1, GALK1, GALT, GCNT2, GFER, GJA3, GJA8, HMX1, HSF4, JAM3, LIM2, MAF, MAN2B1, MIP, MYH9, NF2, NHS, OCRL, OPA3, OTX2, P3H2, PAX6, PEX10, PEX11B, PEX12, PEX13, PEX14, PEX16, PEX19, PEX26, PEX3, PEX5, PEX6, PITX3, PXDN, RAB18, RAB3GAP1, RAB3GAP2, SC5D, SEC23A, SIL1, SLC16A12, SLC2A1, SLC33A1, SLC4A4, SRD5A3, TBC1D20, TDRD7, WFS1, WRN, VIM* |
| Aniridia | Panocular disorder caused by *PAX6* haploid insufficiency from approximately week 5 that affects development of multiple ocular structures. | Iris and foveal hypoplasia, optic nerve anomalies and microphthalmia. Later onset cataract, glaucoma and corneal keratopathy | Week 5-8 | *ELP4, FOXC1, PAX6, PITX2, PXDN*, TRIM44** |
| Corneal hereditary endothelial dystrophy | Primary endothelial cell dysfunction caused by *SLC4A11* variants that ion transportation and cellular osmolarity. | Severe visual impairment caused by bilateral corneal edema characteristic | Week 5-8 | *SLC4A11* |

*****Little evidence suggesting association with disease

**Table 3: Common early developmental eye disorders and associated genes**
